# Paper-Recorded ECG Digitization Method with Automatic Reference Voltage Selection for Telemonitoring and Diagnosis

**DOI:** 10.3390/diagnostics14171910

**Published:** 2024-08-29

**Authors:** Liang-Hung Wang, Chao-Xin Xie, Tao Yang, Hong-Xin Tan, Ming-Hui Fan, I-Chun Kuo, Zne-Jung Lee, Tsung-Yi Chen, Pao-Cheng Huang, Shih-Lun Chen, Patricia Angela R. Abu

**Affiliations:** 1 School of Advanced Manufacturing, Fuzhou University, Quanzhou 362200, China; eetommy@fzu.edu.cn; 2 The Department of Microelectronics, College of Physics and Information Engineering, Fuzhou University, Fuzhou 350108, China; 221110012@fzu.edu.cn (C.-X.X.); yangtao_fzu@fzu.edu.cn (T.Y.); 221127049@fzu.edu.cn (H.-X.T.); 3 College of Biological Science and Engineering, Fuzhou University, Fuzhou 350108, China; t20106@fzu.edu.cn; 4 Department of Electronic Engineering, Feng Chia University, Taichung 40724, Taiwan; tsungychen@fcu.edu.tw; 5 Department of Electronic Engineering, National Cheng Kung University, Tainan 70101, Taiwan; paul0328@gmail.com; 6 The Department of Electronic Engineering, Chung Yuan Christian University, Taoyuan 32023, Taiwan; chrischen@cycu.edu.tw; 7 The Department of Information Systems and Computer Science, Ateneo de Manila University, Quezon City 1108, Philippines; pabu@ateneo.edu

**Keywords:** ECG signal extraction, ECG data recovery, image distortion correction, uneven light correction, signal reconstruction

## Abstract

In electrocardiograms (ECGs), multiple forms of encryption and preservation formats create difficulties for data sharing and retrospective disease analysis. Additionally, photography and storage using mobile devices are convenient, but the images acquired contain different noise interferences. To address this problem, a suite of novel methodologies was proposed for converting paper-recorded ECGs into digital data. Firstly, this study ingeniously removed gridlines by utilizing the Hue Saturation Value (HSV) spatial properties of ECGs. Moreover, this study introduced an innovative adaptive local thresholding method with high robustness for foreground–background separation. Subsequently, an algorithm for the automatic recognition of calibration square waves was proposed to ensure consistency in amplitude, rather than solely in shape, for digital signals. The original signal reconstruction algorithm was validated with the MIT–BIH and PTB databases by comparing the difference between the reconstructed and the original signals. Moreover, the mean of the Pearson correlation coefficient was 0.97 and 0.98, respectively, while the mean absolute errors were 0.324 and 0.241, respectively. The method proposed in this study converts paper-recorded ECGs into a digital format, enabling direct analysis using software. Automated techniques for acquiring and restoring ECG reference voltages enhance the reconstruction accuracy. This innovative approach facilitates data storage, medical communication, and remote ECG analysis, and minimizes errors in remote diagnosis.

## 1. Introduction

Cardiovascular diseases (CVD), which have surpassed the mortality rate of cancer, tend to affect the younger population and have become a huge social burden [1,2]. Given the inevitable uneven distribution of medical resources, a contradiction exists between the scarcity of high-quality physician resources in underdeveloped areas and the overburdened medical capacity in densely populated areas; this phenomenon is reflected in the remarkably high proportion of CVD mortality in less developed areas [3,4,5].

Electrocardiogram (ECG), as a non-invasive approach, is extensively utilized in the diagnosis of cardiovascular diseases [6]. With the penetration of the internet and the advancement of artificial intelligence algorithms, a significant number of researchers have focused on developing algorithms for the diagnosis of cardiovascular diseases based on ECGs, including arrhythmia classification [7,8,9,10], atrial fibrillation detection [11,12], and myocardial infarction detection [13,14,15]. The development of intelligent diagnostic algorithms based on ECGs has, to some extent, enhanced the accuracy of electrocardiographic diagnoses and alleviated the issue of the uneven distribution of medical resources. Furthermore, many researchers are exploring other applications of ECGs, such as biometric identification [16,17,18] and emotion recognition [19,20,21]. Fundamentally, such research requires huge numerical data records with manual labels.

However, the difference in the storage mode of ECG data makes it difficult to ensure high data utilization. The lack of easily available ECG data for research purposes is still a common problem [22]. Tracing back to the source, the uneven allocation of resources and the encryption of patient data lead to the failure of forming a unified communicable ECG storage format, which further leads to the contradiction between the increasingly perfect ECG diagnosis algorithm and the clinical needs of patients. Clinically, most doctors rely on special analysis software that can import the collected electronic documents of patients’ physiological signals to make a diagnosis efficiently and accurately. By contrast, in most cases of data storage systems of community medical institutions and large public hospitals, only medical explanations or at most raster images storing ECG waveforms are retained unless some ECG management systems exist [23,24]. In addition, the degree of information sharing and recognition among hospitals remains in doubt because the archiving of source data information distributed in hospitals in different regions is often scattered in several systems rather than transmitted to the central electronic medical record system.

Paper-recorded ECGs, which are used for data exchange and individual retrospective analysis, remain the main storage form for patients to keep records of their case. Under the premise of obtaining the authorization of patients, paper ECG waveforms must be converted into one-dimensional digital signals and stored as electronic files for the establishment of specific databases and individual risk assessments of long-term heart disease. A method of digitizing paper ECGs, which can be regarded as a bridge for data sharing, is expected. At the same time, patients can also better store the digitized ECG data for long-term use of ECG records in different hospitals, therefore avoiding the problem that the original sampling data cannot be circulated due to encryption.

Many scholars have found a suitable digital method and obtained relevant research results, in which optical scanning is the first representative digital method [25,26,27,28]. Lobodzinski et al. [25] used optical scanning to acquire textual information and waveform images on paper ECGs. Mitra et al. [26] used a flatbed scanner to form an image database of each 12-lead ECG signal. Karsikas et al. [27] used an optical flatbed scanner to digitize the data on paper, and set a threshold to remove the red grid lines from the paper data. Ravichandran et al. [28] used a scanner to digitize paper data and used OCR technology to extract patient-related information from paper ECGs. In recent years, research in this field has been extended to more dimensions [29,30,31]. Hao et al. [29] used a machine learning method to classify diseases from paper ECGs, which can avoid the problem of the original ECG data being difficult to obtain. Kavak et al. [30] used a convolutional neural network to obtain the characteristic information of ECG signals from paper ECGs and complete the location of myocardial infarction. In these methods, standard image data can be obtained by scanning, which can solve the issue of the recognition of some cardiovascular diseases.

However, methods based on optical scanning images are limited by the scanning equipment available, which hinders their widespread application among a large patient population. With the expansion of image sensor areas and advancements in fidelity, cameras embedded in mobile devices now ensure a high degree of digital image restoration. Capturing ECG images using cameras has thus become a more convenient approach, though it also introduces additional challenges in the digitization process.

To convert an ECG image from cameras into amplitude signal data, the process primarily involves three aspects: image distortion correction, binarization, and the digitization of ECG curves [32,33].

In research into distortion correction, Math et al. [34] used Hough transform to calculate the tilt angle of the plane. This method is limited to the tilt correction of the plane image and cannot correct the distortion of the three-dimensional space. For binarization, the methods proposed by different scholars on gridline removal are nearly all based on thresholding [26,28,29,34]. For the digitization of ECG curves, Li et al. [35] proposed a method of converting printed ECG data into digital ECG signals. In this study, for different paper ECGs, we need to redetermine the voltage and time corresponding to the pixel value. However, in recent research into digitization algorithms, almost no one has looked for or attached importance to a suitable reference to ensure the effectiveness of the amplitude voltage [32]. In other words, the current research [35,36,37,38] can only ensure the similarity of the waveform shape, but not the amplitude consistency of the recovered one-dimensional signal.

The contributions of the algorithm proposed in this study can be summarized as the following points:

1. This study departs from the traditional approach of digitizing ECG data based on optical scanning images, instead utilizing more readily accessible camera images for the digitization of ECG data;
2. We have observed the distinctive characteristic of ECGs within the Hue Saturation Value (HSV) color space and have adeptly applied it to the removal of grid lines;
3. The integration of gamma transformation with the OTSU algorithm [39] is introduced, and an innovative adaptive local thresholding algorithm is proposed for the extraction of ECG curves with enhanced precision;
4. An innovative algorithm for the automatic recognition of calibration square waves is proposed to ensure amplitude consistency in the restored signals, rather than merely morphological similarity;
5. The results based on the MIT-BIH normal sinus rhythm database and PTB diagnostic ECG database demonstrate that the proposed method has a higher correlation coefficient than most other methods and can recover the original sampling data perfectly. This algorithm can provide a standard and low-noise input for remote monitoring and diagnosis based on deep learning technology.

The remainder of this paper is organized as follows: Section 2 describes the method of denoising a paper ECG and reconstructing it into the original ECG sampling signal; the evaluation of the experiment results of the reconstruction algorithm and discussion are set out in Section 3; and finally, Section 4 concludes this study.

## 2. Materials and Methods

**Figure 1:**
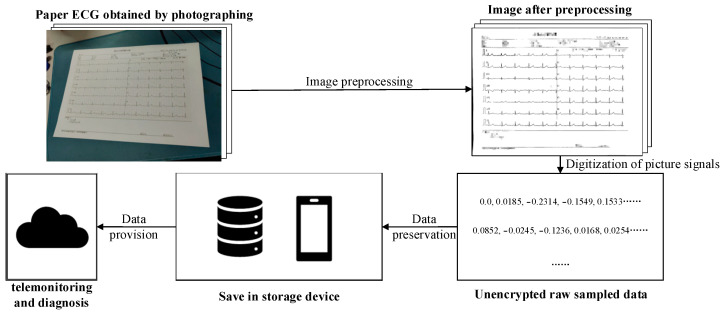
Scheme of paper-recorded ECG digitization system for telemonitoring and diagnosis.

The scheme of the proposed system is shown in Figure 1. This chapter is divided into three research priorities: In the first part, the use of a projection distortion correction algorithm for digital ECG images is proposed with the goal of identifying the four vertices of the ECG waveform from the complex background. The flattened image without geometric distortion is obtained by combining the four coordinates with perspective transformation. Secondly, a targeted improvement of the Otsu algorithm and a postprocessing algorithm are designed to obtain the ECG curve information. This method can effectively solve the problem caused by uneven illumination and considerably improve the success rate of separation in real scenes. After the correct binarization of the image, in the third part, a robust algorithm for converting multi-lead curve 2D images to 1D time-series data is proposed. In this algorithm, the benchmark information is obtained automatically. In contrast to the current research, a novel conversion benchmark is introduced in the digital phase, thereby significantly improving the conversion accuracy.

### 2.1. Adaptive Location and Distortion Correction of ECG

To preprocess the image, it needs resized to 800 px × 600 px and filtered with a Gaussian filter to reduce noise. The edges within the image were detected using the Canny algorithm [40]. Firstly, the gradient magnitude and direction at each point in the image were computed using the Sobel operator, as described by the following equations:

(1) $$G_{x}=\left[\begin{array}{ccc} 1 & 0 & -1\\ 2 & 0 & -2\\ 1 & 0 & -1 \end{array}\right]*A,$$

(2) $$G_{y}=\left[\begin{array}{ccc} 1 & 2 & 1\\ 0 & 0 & 0\\ -1 & -2 & -1 \end{array}\right]*A,$$

where A denotes the original image. The horizontal and vertical gradients at each point in the image are represented by $G_{x}$ and $G_{y}$, respectively. Consequently, the gradient magnitude (i.e., edge strength) and gradient direction (i.e., edge orientation) can be determined as follows:

(3) $$G=\sqrt{G_{x}^{2}+G_{y}^{2}},$$

(4) $$\alpha =\arctan\left(\frac{G_{y}}{G_{x}}\right).$$

Based on the angle α, the edge directions in the image are categorized into eight discrete directions: 0, 45, 90, 135, 180, 225, 270, and 315 degrees, which correspond to the vertical, horizontal, and diagonal directions. Among neighboring points with the same edge direction, only the points with the maximum edge strength are retained.

Subsequently, the low and high threshold parameters are set to 100 and 200, respectively, to determine whether each point is a true edge. Points with an edge strength exceeding the high threshold are considered strong edge points and are preserved. Points with edge strength below the low threshold are deemed non-edge points and are discarded. Points with an edge strength between the low and high thresholds are retained as strong edge points if they are connected to any strong edge points; otherwise, they are discarded. Through these steps, the edges of the image are delineated.

Based on the candidate edges, the Hough Transform algorithm [41] is employed for line detection. A line in the image space (i.e., Cartesian coordinate system) can be uniquely determined by a pair (r, θ) and is represented as:

(5) $$y=-\frac{\cos\theta }{\sin\theta }x+\frac{r}{\sin\theta }.$$

For any point $\left(x_{1},\;y_{1}\right)$, there is a corresponding curve in the parameter space (i.e., polar coordinate system) given by:

(6) $$r=x_{1}\cos\theta +y_{1}\sin\theta .$$

If two points lie on the same straight line, their corresponding curves in the parameter space will converge to a single point  (r, θ). The greater the number of points intersecting at the point $\left(r,\;\theta \right)$, the more points there are in the image space that lie on the straight line determined by $\left(r,\;\theta \right).$ In addition, the θ is evenly divided into 180 intervals. As long as the value $\theta_{1}$ of the intersection point $\left(r_{1},\theta_{1}\right)$ of the curves falls within the interval $\left[\theta_{a},\theta_{a}+\frac{\pi }{180}\right]$, the points in the image space corresponding to the curves in the parameter space are considered to lie on the same straight line. Furthermore, only when more than 100 edge points intersect in the parameter space are these edge points determined to lie on a single straight line. Moreover, the number of intersection points corresponds to the length of the straight edge. The shorter edge other than the two edges with consistent orientation will be removed. The orientation is judged by the cosine of the angle between the straight lines as a standard, as expressed in the formula:

(7) $$\cos\left(l_{1},l_{2}\right)=\left|\frac{x_{1}x_{2}+y_{1}y_{2}}{\sqrt{x_{1}^{2}+y_{1}^{2}}\sqrt{x_{2}^{2}+y_{2}^{2}}}\right|.$$

If the cosine value exceeds 0.85 (i.e., corresponding to an angle of 31.79°), the two lines are deemed to have a consistent orientation. Given that ECG images are approximately rectangular, our goal was to extract the outer vertical and horizontal edges of the ECG image, and any other candidate edges aligned with these needed be removed. Additionally, the edge lines inside the ECG image are mostly parallel to the edges of the ECG. We conducted an experiment involving 20 participants, each capturing 20 images of ECGs from different environments and angles. Our statistical analysis revealed that almost all lines originating from the background formed angles less than 20° (i.e., corresponding to a cosine similarity of 0.94) with the ECG edges. To further enhance the robustness of the proposed algorithm, the cosine value threshold of 0.85 was determined.

Finally, to avoid comparing ECG edges with the same direction (i.e., vertical or horizontal), we divided the image into four comparison regions, namely, top, bottom, left, and right, based on the center of the image. In each region, we determined one final edge, which corresponds to the ECG edge. The intersections of these edge lines were then used to determine the four vertices: bottom-left, top-left, bottom-right, and top-right.

The current detected coordinate position is not the actual vertex position of the original image due to the resizing step in the preprocessing stage. Thus, the four coordinates are converted to the percentage position coordinates in the image and then remapped to the original image so that the original coordinates are finally calculated.

The four two-dimensional coordinate vertices are first introduced and mapped to a three-dimensional space; these vertices are expressed in mathematical formulas as follows:

(8) $$\left[x^{\prime },\;y^{\prime },\;w^{\prime }\right]=\left[u,\;v,1\right]\left[\begin{array}{ccc} a_{11} & a_{12} & a_{13}\\ a_{21} & a_{22} & a_{23}\\ a_{31} & a_{32} & a_{33} \end{array}\right]=\left[u,\;v,1\right]A.$$

The 3 × 3 matrix mentioned above A is the perspective transformation matrix, (u, v) is the original two-dimensional plane coordinates, and (x′ , y′, w′) is the coordinates mapped to the three-dimensional space. The coordinates after the perspective transformation are:

(9) $$x=\frac{x^{\prime }}{w^{\prime }}=\frac{a_{11}u+a_{21}v+a_{31}}{a_{13}u+a_{23}v+a_{33}},$$

(10) $$y=\frac{y^{\prime }}{w^{\prime }}=\frac{a_{12}u+a_{22}v+a_{32}}{a_{13}u+a_{23}v+a_{33}}.$$

Therefore, based on the coordinates of the four vertices, the perspective transformation matrix can be computed, which subsequently allows for the determination of the coordinates (x,y) on the two-dimensional plane. One of the advantages of this algorithm is that it can automatically calculate the correct aspect ratio of the corrected image.

### 2.2. ECG Curve Extraction Algorithm

The focus of this section is on how to separate the curve of the ECG from the image. The aim is to retain the effective original ECG information, including the ECG waveform curves and ECG lead markers while filtering out the background grid and other text records which are significant data to which doctors refer for their diagnoses. The challenges of this lie in the noise caused by uneven illumination, color deviation, and noise in real scenes which constitute the main discussion of this section.

The success of the image separation algorithm depends on the selection of the threshold value. However, after analyzing the histogram after directly graying the red–green–blue (RGB) color mode image in the same scale, the gray difference between the curve and the background grid is not evident, which may lead to the failure of separation caused by many meshes or noises. The color adjustment layer added before the curve extraction in this section focuses on widening the difference between the curve and the grid. The algorithm introduces the use of the hue saturation value (HSV) color space transformation to realize the enhancement algorithm that can adjust the saturation of the specified hue. Compared with adjusting the values directly in the RGB space, one of the advantages of adjusting the values in the HSV color space is that the distribution range of colors can be quantified concretely. In addition, the RGB space is in a color space with significant adjustment limitation because it consists of three discrete channels.

**Figure 2:**
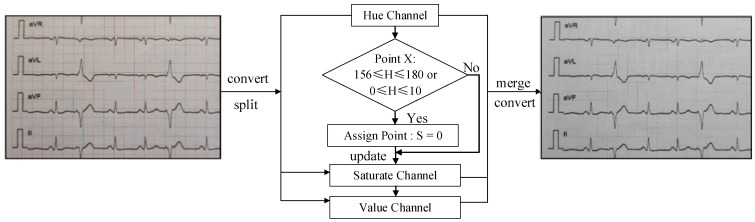
Targeted color adjustment algorithm: the original ECG image is converted and split into hue (H), saturate (S), and value (V) channels. The difference between the grid and the ECG curves is expanded at the most moderate level.

The hue and saturate channels in the HSV color space are used in the proposed algorithm. The lower and upper limits of the hue channel range are 0° and 180°, respectively, which are connected end-to-end in a ring. The ECG images confirm that the red grid to be adjusted corresponds to the hue channel between the [156, 180] and [0, 10] intervals [42] in which all points would be identified. To remove the grid, pixels with hue values within the interval can be converted to white by setting their saturation to zero. The completed process is illustrated in Figure 2. 

It is important to note that the hue channel range [156, 180] and [0, 10] specified is tailored for red grids, which are the most common grid color in ECG images. If the grid is of a different color, the hue channel range should be adjusted accordingly. For example, blue grids correspond to a hue range of [100, 124].

To further isolate the electrocardiographic curve from the image, this study proposed an improved OTSU algorithm. The OTSU algorithm automatically determines the optimal threshold value by maximizing the between-class variance, which simplifies the thresholding process and eliminates the need for manual intervention [39]. It has been widely adopted in various fields [43,44,45].

Assuming an image of size  M×N with an optimal binarization threshold T that divides the image into foreground and background regions. The number of pixels in the foreground and background is $N_{0}\;$ and $N_{1}$, respectively, with proportions of $\omega_{0}$ and $\omega_{1}\;$ (i.e., $1-\omega_{0}$) relative to the entire image. The mean grayscale values of the foreground and background are $\mu_{0}$ and $\mu_{1}$, respectively. Additionally, the mean grayscale value of the entire image is μ, which can be expressed as:

(11) $$\mu =\frac{\mu_{0}\times N_{0}+\mu_{1}\times N_{1}}{M\times N}=\mu_{0}\omega_{0}+\mu_{1}\omega_{1}.$$

The between-class variance of the background and foreground, which is the critical component of the OTSU algorithm, is computed using the following formula:

(12) $$\sigma^{2}=\omega_{0}{\left(\mu_{0}-\mu \right)}^{2}+\omega_{1}{\left(\mu_{1}-\mu \right)}^{2}.$$

Alternatively, it can be expressed as:

(13) $$\sigma^{2}=\omega_{0}\omega_{1}{\left(\mu_{0}-\mu_{1}\right)}^{2}.$$

The larger the between-class variance, the greater the difference in gray-level distributions between the foreground and background. The OTSU algorithm iterates through each value from 0 to 255, treating each as a potential threshold to calculate the between-class variance. It ultimately outputs a threshold that maximizes the between-class variance. This threshold is regarded as the optimal threshold to separate the foreground from the background.

However, the traditional OTSU algorithm is not suitable for ECG images obtained through cameras due to its global thresholding approach, which assumes a uniform distribution of pixel intensities. To overcome this limitation, the proposed algorithm divides the image into 4 × 4 equal parts and applies a gamma transform [46] to stretch the low-gray region and compress the high-gray region. 

The gray value range of most real images is analyzed, after which a conclusion is drawn. No very low or very high gray value is found in the real image, and its concentration range is in the middle and low gray areas. Such concentrated gray distribution can easily lead to the reduction of fault tolerance of the segmentation threshold. Stretching the peak position of the foreground and background classes is one approach to improve this method. Therefore, the algorithm adds a gamma transform before binarization to achieve a nonlinear grayscale transformation. The transformation can be summarized as follows:

(14) $$g_{out}=A\cdot {g_{in}}^{\gamma },$$

where *A* is utilized to adjust the overall brightness of the output image. The value γ used in this study is 0.8.

**Figure 3:**
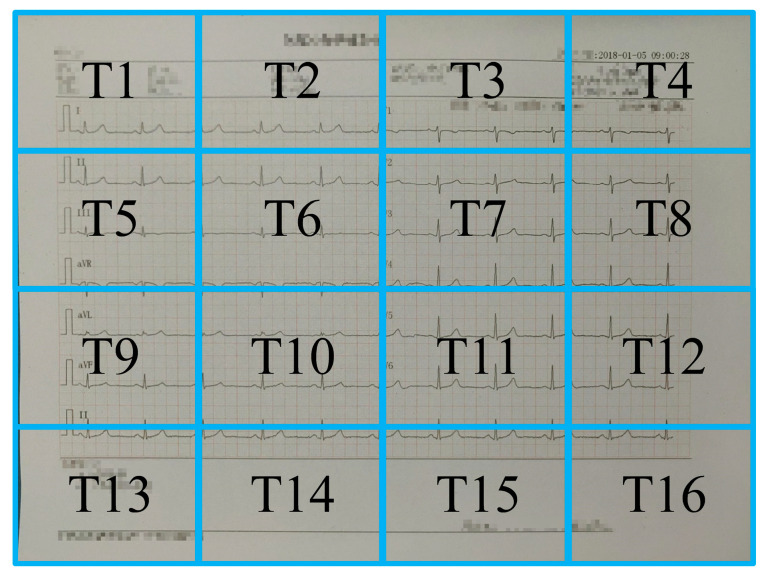
The image was divided into 4 × 4 equal parts and each part has a local threshold value.

Among them, $g_{in}$ and $g_{out}$ are the input and output gray values and $g_{in}$ with the range in [0, 1]. After the normalization of the gray matrix, the gamma transform is conducted, and the gray matrix is remapped into a new grayscale matrix. Finally, 16 image segments are processed using the OTSU algorithm to obtain local threshold values T1–T16, as shown in Figure 3. After each region has been independently binarized, the resulting binary images are stitched to produce a complete binarized ECG image.

Following the binarization process, the regions within the image that possess a grayscale value of 0 correspond to the electrocardiographic curve. In addition, the skeleton thinning algorithm [47] is used to refine the curve to 1 pixel.

### 2.3. The Digitization of ECG Curve

Column-wise scanning is employed to extract the curve information, resulting in a two-dimensional array Arr  that records the coordinate positions of each point on the ECG curve.

Due to ECG signals being output in a specific layout format, the ECG signal of each lead has a fixed position on the ECG report sheet, making it feasible to predefine the coordinates for extraction. In the ECG sheet, it is divided into six leads on the left-half plane (i.e., I, II, III, aVR, aVL, aVF) and six leads on the right-half plane (i.e., V1, V2, V3, V4, V5, V6) which is separated by a vertical line in the middle of the signal, and an additional II-lead at the bottom. Since the acquisition time is fixed (e.g., 5 s for each lead), the complete segment of the signal was reconstructed and subsequently allocated to the respective leads. To avoid data confusion, a small portion of data (i.e., only 0.1 s) at the critical boundary position was discarded.

Additionally, our strategy involves scanning from bottom to top in columns. Text labels are positioned above the effective ECG curves, ensuring that the first detected valid point is from the ECG curve rather than the text label.

**Figure 4:**
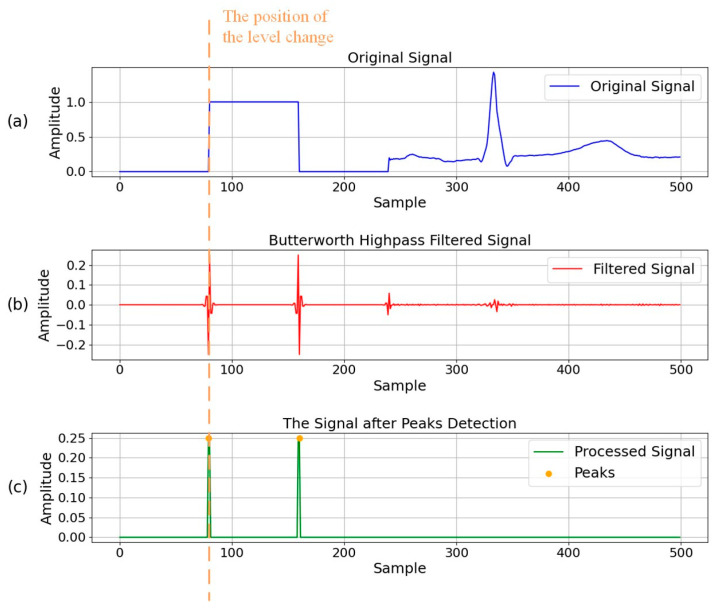
The recognition of the reference square wave based on the Butterworth filter. (**a**) The original signal; (**b**) The signal after second-order Butterworth high-pass filter with a cutoff frequency of 100 Hz; (**c**) The signal after threshold processing and peak detection.

The array contains two main parts: the reference square wave signal and the ECG signal. The former is used as the reference source for the latter to convert into the original ECG amplitude sequence. This study employs a second-order Butterworth high-pass filter with a cutoff frequency of 100 Hz to identify the square wave components within the signal. The frequency range of ECG signals is approximately 0.05–100 Hz. Consequently, after applying a high-pass filter with a cutoff frequency of 100 Hz, the ECG signal is significantly suppressed, while the higher frequency signal at the transitions of square waves is retained, as illustrated in Figure 4b. For a square wave with a high level of 1 mV, the waveform produced after high-pass filtering exhibits a peak amplitude of about 0.25 mV. By taking the absolute value of the filtered signal and setting amplitudes below 0.2 mV to zero, the signal depicted in Figure 4c was obtained. Moreover, the peaks of this signal coincide with the transition points of the square wave levels. The durations of the high level and the low level in the square wave are equal. Therefore, according to the high-level width of the square wave, the starting recording point of the physiological data is at the end point of the square wave signal. Ultimately, an ECG signal data, denoted as Dat, can be acquired subsequent to the exclusion of the square wave component from the array Arr. 

It is important to note that due to variations in image resolution, both the high level of the square wave and the peak amplitude of the filtered waveform will be proportionally scaled. Consequently, the threshold for peak detection must be adjusted accordingly.

**Figure 5:**
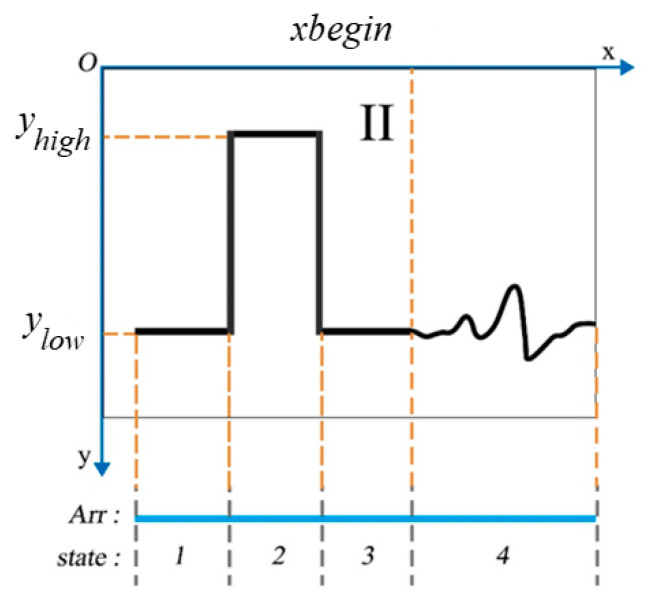
Schematic of benchmark square pulse. The states 1, 2, 3, and 4 refer to the first low-level voltage segment, the high-level voltage segment, the second low-level voltage segment of the square wave, and the ECG signal segment, respectively.

The position and significance of the key points of the square wave and the signal are illustrated in Figure 5. The most important information from a square wave is the difference between its high-level and low-level voltage coordinates in the image. It represents the actual recorded standard voltage difference of 1 mV (the sensitivity is ×1 level), and the value of this ordinate difference is also in effect equal to the abscissa width of the sampling duration of 0.4 s (the paper speed is 25 mm/s). Should there be an alteration in the sensitivity of the calibration square wave or the paper speed of the electrocardiogram, it is imperative that the corresponding computational formulas be scaled proportionately.

Thus, the amplitude conversion ratio θ between the *Y*-axis pixel and the actual voltage amplitude of the image and the sampling conversion ratio β between the *X*-axis pixel and the actual sampling time length can be calculated as:

(15) $$\theta =\frac{1}{\Delta }\;\mathrm{mV}/\mathrm{pixel},$$

(16) $$\beta =\frac{0.4}{\Delta }\;s/\mathrm{pixel},$$

(17) $$\Delta =\left|y_{high}-y_{low}\right|,$$

where $y_{high}$ and $y_{low}\;$ denote, respectively, the ordinate values corresponding to the high and low voltage levels of the square wave. Additionally, the abscissa value at the initial point of the signal is designated as $\;x_{begin}$, and the amplitude of the signal at the low voltage points of the square wave is recorded as 0 mV.

Subsequently, the coordinate values, $\;x_{i}$ and $y_{i}$, within Dat  can be iteratively converted into actual ECG signal amplitudes using the following formula:

(18) $$y_{i}^{\prime }=-\theta \left(y_{i}-y_{low}\right),$$

(19) $$x_{i}^{\prime }=\beta \left(x_{i}-x_{begin}\right).$$

The *Y*-axis direction of the image coordinate system is vertical and downward, which is opposite to the direction of the voltage amplitude from low to high. Thus, the number of the result as the actual voltage should be opposite.

Finally, Table 1 summarizes the steps and parameters of the proposed approach.

## 3. Results and Discussion

### 3.1. Results for Image Distortion Correction

**Figure 6:**
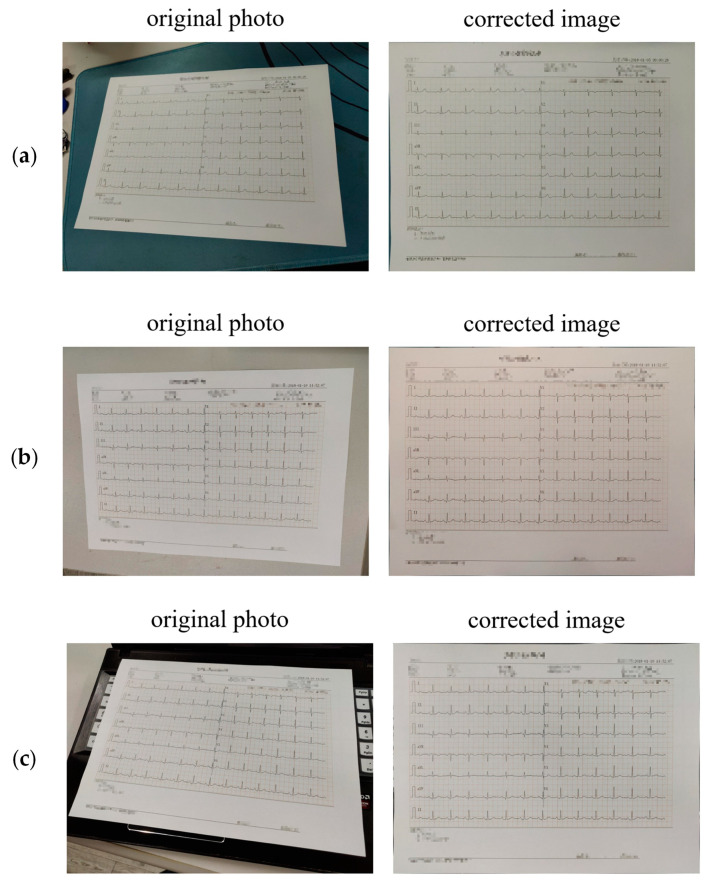
Results for geometric distortion correction. (**a**) ECG photo taken at random; (**b**) ECG photo taken with white background; (**c**) ECG photo taken in a complex background.

The algorithm tests the effect of distortion correction in different shooting environments and backgrounds and selects a representative to show the effect of the algorithm from Figure 6a–c. In Figure 6a, the algorithm takes the images taken in the monotonous environment as the extraction object. The example in this shooting scene can be used as the embodiment of the performance of the correction algorithm in the general background. Figure 6b shows an ECG drawing immersed in a pure white background, which may result in a lack of edge detection. However, the algorithm in this study also corrects the drawing well. Figure 6c is used as the verification of ECG drawing interception and correction under a complex background. The outer outline of the drawing is connected to many invalid edges and some of the edges are connected to the white part of the background, leading to redundancy or missing contours. Nevertheless, the algorithm still accurately identifies the four vertices of the drawing and corrects them, thereby demonstrating the robustness of the core vertex search algorithm. 

Combining the experiments, this study obtained the following conclusions: Unless under extreme shooting conditions, the algorithm can identify well the ECG thermal paper in a scene where the overall brightness of the image is dim or bright. The accuracy of the data after distortion correction is still a content of quantitative analysis in the experiment, which makes a more detailed analysis and a more quantitative conclusion in the third section of this chapter.

### 3.2. Results for ECG Image Binarization

**Figure 7:**
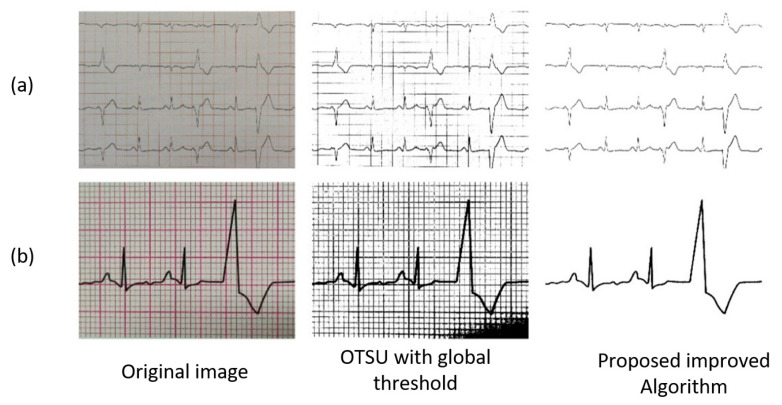
Results for ECG image binarization. The image is taken in different environments. (**a**) photo of common ECG paper saved in patient; (**b**) photo taken from a cardiology book.

**Figure 8:**
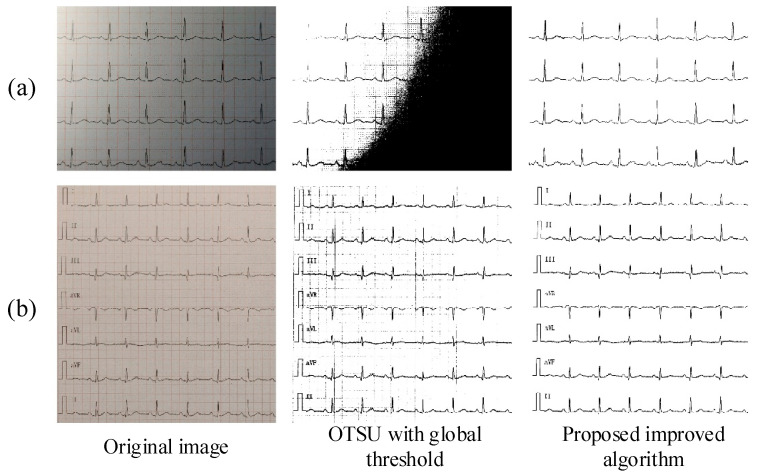
Results for ECG image binarization. The image is taken in different environments. (**a**) photo with strong uneven illumination interference; (**b**) photo of clinical 12-lead ECG thermal paper.

Figure 7 and Figure 8 show that the original images captured by mobile devices in four different cases are used as experimental objects for the extraction of representative curve images in a specific environment. In the group picture in Figure 7a, a multi-lead ECG has a bad effect under the binarization method of the global threshold because the foreground is too close to the background after it is grayed out. This situation becomes even more evident in the single-lead ECG of the group picture in Figure 7b. Nevertheless, this problem can be easily solved by the algorithm proposed in this study because the algorithm itself can stretch the relative difference between the curve and the other parts.

In addition, Figure 7b and Figure 8a show that the image loses information due to having a weak or strong uneven light distribution after binarization. The phenomenon of image loss in Figure 8a is the most serious case since it was specially photographed in an environment with uneven light. The algorithm proposed in this study, which is capable of independently obtaining local separation thresholds for both high-brightness and low-brightness regions in images, can correctly extract the curves in the inhomogeneous illumination image shown in Figure 8a. Figure 8b is a special group because it is part of the complete 10 s clinical ECG thermal paper captured by the mobile phone after distortion correction. The result after OTSU with global threshold processing had many background grids that were mistakenly regarded as part of the ECG curve, which is disastrous for subsequent digitization algorithms. The proposed algorithm can effectively extract the correct and complete multi-lead ECG curve with a pure background.

The performance of the above experimental results can prove that the binarization algorithm in this study is a robust and effective algorithm for ECG curve extraction in real scenes and is regarded as a key link in the digitization of ECG signals.

### 3.3. Results for ECG Image Digitization

To verify the general applicability of the algorithm, the MIT-BIH Normal Sinus Rhythm Database [48] and PTB Diagnostic ECG Database [48,49] were selected as the source of the test dataset, and the data were printed into paper ECG according to the hospital ECG format. From the MIT-BIH Normal Sinus Rhythm Database, we took 15 records, for which the sample rate is 128 Hz. From the PTB Diagnostic ECG Database, we selected data according to different cardiovascular disease categories, and we selected 4–5 records for each type of disease from different patients randomly. In this database, the sample rate is 1000 Hz. For each record, we randomly intercepted 10 s of data for the experiment.

**Figure 9:**
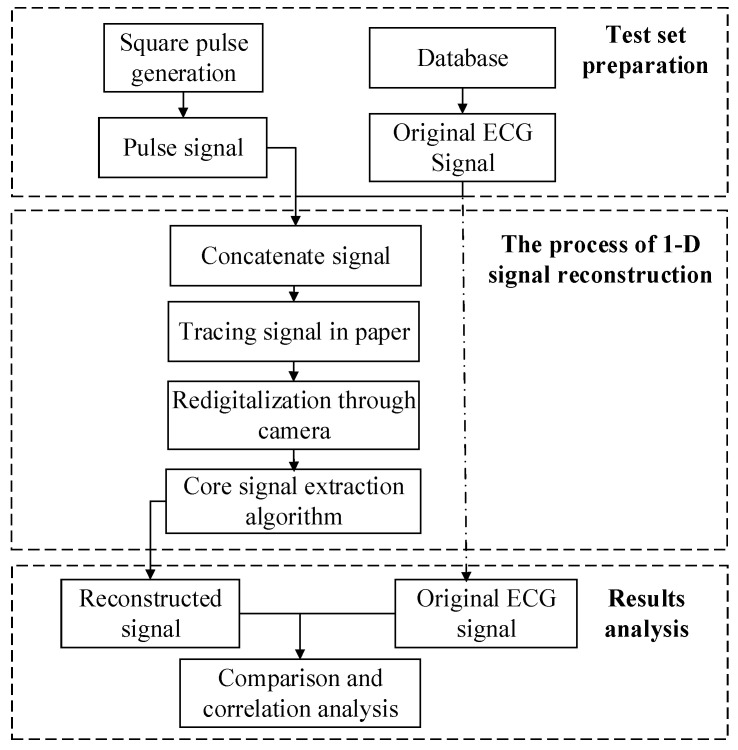
Validation scheme for 1D ECG signal reconstruction algorithm.

Given that the original sampling data of ECG paper are usually archived as an electronic version, the original physiological signals cannot be verified. Therefore, we simulated the entire digitization process including printing the original data on paper to measure the performance of our digital algorithm. The overall design is presented in Figure 9. The validation process can be divided into three stages. In the first part of the test set preparation, a reference square wave generator is designed to have the ECG to print the raw data into a realistic common ECG data pattern. The difference between the high and low level of the generator is 1 mV. Given that the width of the square wave does not contain the reference information, its width is designed as 0.2 s. In the second module, the generated square wave signal and the original ECG signal are concatenated and traced on paper. Moreover, ECG images captured by a camera are reconstructed into digital signals using the algorithm proposed in this study. Finally, the reconstructed signal is compared to the original signal, and a correlation analysis is conducted.

**Figure 10:**
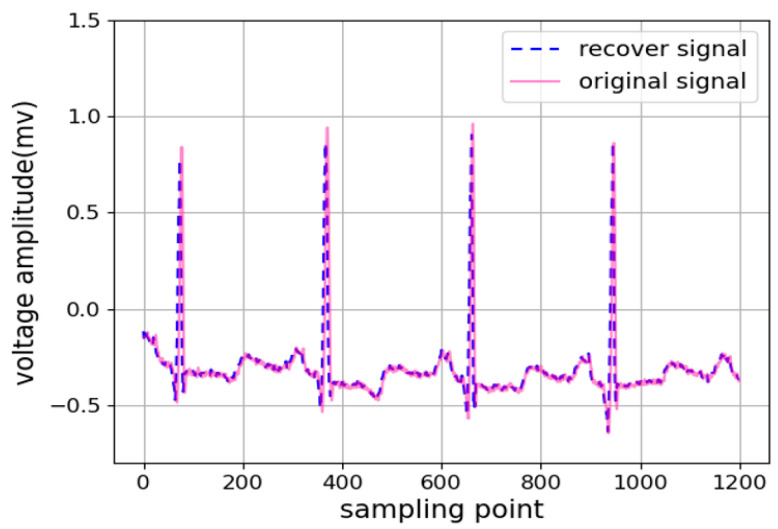
Result of comparison between reconstruction signal and original signal.

Figure 10 shows a sample comparison of waveforms which intuitively show the actual difference between the recover signal represented by the purple dashed line and the original signal represented by the pink line. The difference between the two is hardly noticeable and the consistency is high.

To quantify this difference further, the time-lagged cross-correlation method with the Pearson coefficient as the correlation coefficient is introduced as the correlation verification method. If the elements of original signal and recovered signal are named x and y, then the Pearson coefficient R is expressed as:

(20) $$r=\frac{\sum_{i=1}^{n}\left(x_{i}-\bar{x}\right)\left(y_{i}-\bar{y}\right)}{\sqrt{\sum_{i=1}^{n}{\left(x_{i}-\bar{x}\right)}^{2}}\sqrt{\sum_{i=1}^{n}{\left(y_{i}-\bar{y}\right)}^{2}}},$$

where n is the sequence length, i represents the element in the data sequence, and $\bar{x}$ and $\bar{y}$ denote the average values. 

The ECG Pearson correlation coefficients are listed in Table 2 and Table 3. The overall mean timing similarity reaches 0.97 and 0.98, which implies an excellent performance for the ultralong original ECG sequence and stable performance on different data sets. Additionally, the mean absolute error (MAE) metric was used to quantify the similarity, with the average MAE values for the MIT-BIH Arrhythmia Database and the PTB Diagnostic ECG Database being 0.0324 and 0.0241, respectively. This error corresponds to approximately one-third or one-fourth of a small grid (1 mm × 1 mm) on an ECG.

Table 4 lists a comparison of the correlation values of the current research with those in other literature. The proposed algorithm is better than the results in most of the literature. All the above analysis data indicate that our digitization method is a data reconstruction algorithm with a high degree of recovery and minimal fluctuation of timing and amplitude.

Based on the aforementioned experiments and results, it is evident that the proposed digitization algorithm for paper-based ECGs demonstrates high robustness and accuracy. This advancement facilitates the integration of state-of-the-art intelligent diagnostic algorithms into paper-based ECGs, while also offering new methods for the storage and integration of valuable ECG data.

While our approach has demonstrated outstanding performance in digitizing paper-recorded ECGs, several limitations remain to be addressed. Firstly, the processes of image distortion correction and binarization still partially depend on the quality of the captured photographs. In general, photographs taken in bright, evenly lit, and unobstructed environments are more amenable to image correction and binarization, ultimately yielding better digitized ECG signals compared to those taken in dim or unevenly lit conditions. Additionally, the algorithm requires prior determination of the positions of each lead signal within the ECG image, which is necessary for image segmentation to individually recover each lead’s information. Although the positions of each lead produced in each hospital are typically consistent, we aim to investigate more robust methods in the future to automatically determine the positions of each lead signal in the image, thereby accommodating a wider variety of ECG images.

## 4. Conclusions

This study aimed to convert paper-based records of ECG curves into digital signals to provide standardized, low-noise inputs for remote monitoring and diagnostic systems based on deep learning technology. For the image quality, the study conducted research considering the main concerns and proposing more suitable techniques such as image distortion correction, an image separation algorithm of pure curve, 1D data extraction, and original data reconstruction. The improved Otsu algorithm was used to effectively separate the ECG waveform from the complex background. In the process of digital transformation, an algorithm for the automatic extraction of benchmark information was proposed. This algorithm can greatly improve the accuracy of the ECG waveform conversion. The entire algorithm was validated on the MIT-BIH database and the PTB database and the results were compared in terms of the two aspects of overall sequence validity and physiological data reconstruction analysis. For the correlation analysis of the entire sequence, an average Pearson coefficient of 0.975 was still obtained. Given the results above, the overall reconstruction algorithm achieved high accuracy and robustness.

## Figures and Tables

**Table 1:** Steps and parameters of proposed approach.

Step	Name	Value/Description
Input	ECG images	Obtained through cameras
1	Canny edge detection	Low threshold: 100 High threshold: 200
2	Hough line transform	Angular resolution: π/180 Threshold: 100
3	ECG edge lines decision	The shorter line was removed Cosine threshold: 0.85
4	Four vertices detection	The intersection point of lines
5	Image distortion correction	Based on perspective transformation
6	Red gridlines removal	Based on the HSV color space Hue range: [0, 10], [156, 180]
7	Image segmentation	4 × 4 equal parts
8	Gamma transformation	γ: 0.8
9	OTSU algorithm	Applied to each of 16 parts
10	Image stitching	Not applicable
11	Column-wise scanning	To extract the curve information
12	Reference square wave detection	Butterworth high-pass filter Order: 2 Cutoff frequency: 100 Hz
13	Amplitude transformation	Based on reference square wave
Output	Digitized ECG amplitude signal	Not applicable

**Table 2:** Similarity analysis between extracted 1D signal value and real value of MIT-BIH.

Record	Pearson r	MAE
16,265	0.980	0.0316
16,272	0.975	0.0218
16,273	0.942	0.0574
16,420	0.978	0.0369
16,483	0.983	0.0281
16,539	0.969	0.0385
16,773	0.980	0.0257
16,786	0.969	0.0436
16,795	0.977	0.0277
17,052	0.969	0.0291
17,453	0.971	0.0215
18,177	0.977	0.0334
18,184	0.952	0.0416
19,088	0.955	0.0305
19,090	0.971	0.0188
AVE	0.970	0.0324

**Table 3:** Similarity analysis between extracted 1D signal value and real value of PTB diagnostic ECG database.

Category of Disease	Record	Pearson r	MAE
Bundle branch block	s0364lre	0.982	0.0148
	s0404lre	0.984	0.0329
	s0421_re	0.951	0.0504
	s0424_re	0.986	0.0303
Cardiomyopathy	s0189_re	0.999	0.0040
	s0200_re	0.984	0.0252
	s0392lre	0.988	0.0213
	s0420_re	0.980	0.0119
Dysrhythmia	s0018cre	0.983	0.0295
	s0169_re	0.992	0.0095
	s0211_re	0.970	0.0309
	s0349lre	0.996	0.0103
	s0393lre	0.980	0.0191
healthy	s0291lre	0.961	0.0344
	s0303lre	0.978	0.0251
	s0306lre	0.987	0.0267
	s0311lre	0.950	0.0330
AVE		0.980	0.0241

**Table 4:** Comparison of current research in terms of correlation.

Method	Average Value of Overall Correlation
Ravichandran’s algorithm [28]	0.85–0.90
Baydoun’s algorithm [50]	0.952
Gautam’s algorithm [37]	0.86
Li’s algorithm [35]	0.916
This work (AVE)	0.975

## Data Availability

The MIT-BIH Normal Sinus Rhythm Database is available at https://www.physionet.org/content/nsrdb/1.0.0/ (accessed on 4 August 2020); PTB database is available at https://www.physionet.org/content/ptbdb/1.0.0/ (accessed on 4 August 2020).
